# Consensus guidelines for diagnosis, treatment and follow-up of patients with pancreatic cancer in Spain

**DOI:** 10.1007/s12094-016-1594-x

**Published:** 2016-12-19

**Authors:** M. Hidalgo, R. Álvarez, J. Gallego, C. Guillén-Ponce, B. Laquente, T. Macarulla, A. Muñoz, M. Salgado, R. Vera, J. Adeva, I. Alés, S. Arévalo, J. Blázquez, A. Calsina, A. Carmona, E. de Madaria, R. Díaz, L. Díez, T. Fernández, B. G. de Paredes, M. E. Gallardo, I. González, O. Hernando, P. Jiménez, A. López, C. López, F. López-Ríos, E. Martín, J. Martínez, A. Martínez, J. Montans, R. Pazo, J. C. Plaza, I. Peiró, J. J. Reina, A. Sanjuanbenito, R. Yaya, Alfredo Carrato

**Affiliations:** 1 Spanish National Cancer Centre, C/Melchor Fernández Almagro, 3, 28029 Madrid, Spain; 2 Beth Israel Deaconess Medical Center, Boston, USA; 3 Department of Medical Oncology, Centro Integral Oncológico Clara Campal, Madrid, Spain; 4 University Hospital of Elche, Elche, Spain; 5 Hospital Universitario Ramón y Cajal, Ctra. de Colmenar Viejo km. 9,100, 28034 Madrid, Spain; 6 Institut Català d´Oncologia, Duran y Reynals Hospital, Hospitalet Llobregat, Barcelona, Spain; 7 Vall d’Hebrón University Hospital, Barcelona, Spain; 8 University Hospital Gregorio Marañón, Madrid, Spain; 9 University Hospital of Ourense, Ourense, Spain; 10 Complejo Hospitalario de Navarra, Pamplona, Spain; 11 University Hospital 12 de Octubre, Madrid, Spain; 12 Hospital Carlos Haya, Málaga, Spain; 13 University Hospital Donostia, San Sebastián, Spain; 14 Department of Radiology, University Hospital Ramón y Cajal, Madrid, Spain; 15 MD Anderson Hospital, Madrid, Spain; 16 Department of Palliative Care, Hospital Germans Trias I Pujol, Institut Catalá d´Oncologia, Badalona, Spain; 17 Department of Medical Oncology and Hematology, University Hospital Morales Messeguer, Murcia, Spain; 18 Department of Gastroenterology, Hospital General Universitario de Alicante, Alicante, Spain; 19 Department of Medical Oncology, Hospital Universitari I Politècnic La Fe, Valencia, Spain; 20 Department of Surgery, Hospital Clínico San Carlos, Madrid, Spain; 21 Department of Medical Oncology, Hospital Son Llàtzer, Palma de Mallorca, Spain; 22 Hospital Clínico San Carlos, Madrid, Spain; 23 Complejo Hospitalario Universitario de Pontevedra, Pontevedra, Spain; 24 Complejo Hospitalario de Granada, Granada, Spain; 25 Department of Radiotherapy, University Hospital HM Sanchinarro, Madrid, Spain; 26 University Hospital HM Puerta del Sur, Madrid, Spain; 27 Department of Medical Oncology, Hospital Universitario Central de Asturias, Asturias, Spain; 28 Hospital Universitario de Burgos, Burgos, Spain; 29 Hospital Universitario Marqués de Valdecilla, Santander, Spain; 30 Department of Pathology, University Hospital HM Sanchinarro, Madrid, Spain; 31 Department of Surgery, Hospital Universitario de la Princesa, Madrid, Spain; 32 Department of Medical Oncology, University Hospital Virgen de las Nieves, Granada, Spain; 33 Hospital del Mar, Barcelona, Spain; 34 Department of Pathology, Centro Anatomopatológico, Madrid, Spain; 35 Department of Medical Oncology, University Hospital Miguel Servet, Saragossa, Spain; 36 Department of Endocrinology, Instituto Catalán de Oncología, Hospital Duran I Reynals, Hospitalet de Llobregat, Barcelona, Spain; 37 Department of Medical Oncology, University Hospital Virgen de la Macarena, Seville, Spain; 38 Department of Surgery, University Hospital Ramón y Cajal, Madrid, Spain; 39 Department of Medical Oncology, Fundación Instituto Valenciano de Oncología, Valencia, Spain

**Keywords:** Pancreatic cancer, Diagnosis, Treatment, Consensus guidelines

## Abstract

The management of patients with pancreatic cancer has advanced over the last few years. We convey a multidisciplinary group of experts in an attempt to stablish practical guidelines for the diagnoses, staging and management of these patients. This paper summarizes the main conclusions of the working group. Patients with suspected pancreatic ductal adenocarcinoma should be rapidly evaluated and referred to high-volume centers. Multidisciplinary supervision is critical for proper diagnoses, staging and to frame a treatment plan. Surgical resection together with chemotherapy offers the highest chance for cure in early stage disease. Patients with advanced disease should be classified in treatment groups to guide systemic treatment. New chemotherapeutic regimens have resulted in improved survival. Symptomatic management is critical in this disease. Enrollment in a clinical trial is, in general, recommended.

## Introduction

Pancreatic ductal adenocarcinoma (PDAC), the most common form of pancreatic cancer, currently stands as the third most common cause of cancer related deaths [1]. In 2008 a total of 70,000 of were diagnosed in Europe [2, 3]. Rates of new cases of pancreatic cancer have increased on average 0.8% annually over the last ten years. The current 5- and 10-year survival rates at 7.2% and below 4%, respectively [4].

The aim of the present consensus guidelines is to provide a general overview of the diagnosis, a more global patient´s classification, not just based on performance status, treatment, and management of associated complications of patients with PDAC. These guidelines are the result of expert consensus meetings that took place during the months of September to December 2015 sponsored by Fundación ECO, where a total of forty-two medical oncologists, radiotherapists, surgeons, radiologists, pathologists, endocrinologists, gastrointestinal specialists and palliative care specialists shared their opinions. These recommendations are based on the results of clinical trials, retrospective, observational studies, as well as the group of experts´ opinion (levels of evidence: quality of evidence: I–III; strength of recommendation: A–E) [5].

## PDAC: signs and symptoms

The absence of specific manifestations, together with its biological aggressiveness, results in delayed diagnosis in more than 80% of cases. The most common symptoms include fatigue, anorexia, weight loss, abdominal pain and dark urine [6]. Sixty to 70% of tumors originate in the pancreatic head, 20–25% in the body and tail and in 10–20% there is a diffuse involvement of the gland [7]. While tumors in the pancreatic head tend to be diagnosed at earlier stages because of the jaundice associated with bile duct obstruction, tumors in the body and tail are usually detected in advanced stages. Tumor with obstructive jaundice may be associated with palpable gallbladder (Courvoisier sign). Head tumors may also be associated with steatorrhea as a consequence of exocrine pancreatic insufficiency and obstruction of the pancreatic duct. Although with low specificity, the combination of diabetes mellitus (DM) of recent onset associated with weight loss should lead to suspicion PDAC [8]. About one in 125 (0.85%) patients with new-onset DM presents PDAC, eight times more than expected, compared to the general population [9]. Patients with newly diagnosed DM without metabolic syndrome and difficult glycemic control should be evaluated to rule out PDAC [10] (IIB). Special attention should be paid to patients that present the following symptoms: weight loss, abdominal pain, DM of recent onset or with poor disease control. Patients with advanced stages of PDAC may have epigastric palpable mass, hepatomegaly secondary to liver metastases, ascites caused by peritoneal carcinomatosis, pain, vascular and nerve infiltration and/or gastric outlet obstruction. The PDAC is associated with arterial and venous (hypercoagulable state) thrombosis such as Trousseau syndrome (migratory superficial vein thrombosis. PDAC may also be associated with palpable supraclavicular lymph nodes (Virchow), anterior axillary (Irish) or periumbilical mass (Sister Mary Joseph).

## Familial PDAC and hereditary PDAC

The International Cancer of the Pancreas Consortium Screening (CAPS) recommends screening of PDAC in high-risk individuals (HRI) or families with familial pancreatic cancer (FPC), although this is not yet included in routine clinical practice [11] (IIIC). PDAC screening programs should be performed in high-volume centers that have multidisciplinary teams with experience in the screening and treatment of the disease. HRI are considered as those who have at least 5–10 times increased risk of PDAC [11] and include families with at least two first-degree relatives affected, or ≥3 individuals regardless of the degree of kinship. In addition, subjects with Peutz–Jeghers syndrome, hereditary pancreatitis, or those affected by other hereditary cancer syndromes (germline mutations in the BRCA genes, familial melanoma or colorectal cancer hereditary) with a case of PDAC in the family [12].

The best screening techniques for HRI is magnetic resonance imaging (MRI) with magnetic resonance cholangiopancreatography (MRCP) and upper gastrointestinal endoscopic ultrasound (EUS) [11–15] (Algorithm 1). Screening tests aim to detect small pancreatic solid tumors (≤1 cm), irregular pancreatic duct, and pancreatic precursor lesions such as intraductal papillary mucinous neoplasm (IPMN), which are usually present as cysts [16]. The prevalence of suspected cystic pancreatic lesions is 33–45% in HRI [11, 15]. The CAPS recommend starting screening at age 50 years, or ten years before the age of the youngest case in the family [11] with annual exams if no pancreatic lesions are observed [11] (IIIB). These intervals could be modified at the discretion of the multidisciplinary tumor committee. If suspicious abnormalities are detected, surgery is indicated although the current available evidence is limited [11–16].

## Diagnosis

In patients with suspected PDAC based on medical history and/or physical exam, the primary diagnostic approach to the patient with PDAC is radiological. An abdominal ultrasound is often the first test performed in patients with abdominal pain and/or jaundice. US can detect dilatation of the bile duct or pancreatic duct and the presence of a pancreatic mass. However, the sensitivity for detecting pancreatic tumors is low and ranges between 50 and 70% [17]. Computer tomography (CT) scan is the gold standard technique in the evaluation of patients with suspected PDAC (IIA). The study of a pancreatic mass must include a biphasic CT performed with an arterial phase (40–50 s) and portal venous phase (65–70 s) [7, 17–19]. In most cases, PDAC is seen in the arterial phase as a hypodense pancreatic lesion, with poorly defined margins [7]. Furthermore, CT scan detects the vascular involvement (arterial and venous), extra-pancreatic local extension, presence of lymph nodes, and detection of liver or peritoneal nodules [7]. The availability of a high-quality multidetector CT (preferably ≥16 detectors) combined with experience in the interpretation of these studies has shown a more accurate preoperative staging and better patients´ management [18]. Magnetic resonance imaging (MRI) has equal sensitivity and specificity than CT for staging PDAC, but its use is not widespread because of its high cost and reduced availability and is usually reserved for difficult cases, cystic neoplasms of the pancreas and to explore biliary anatomy as well [18]. Radiology report should follow a standard format [18, 19].

Upper gastrointestinal endoscopy is useful to take biopsies of tumors infiltrating the duodenum as well as for palliative decompression of the duodenum and/or bile duct [7]. Endoscopic retrograde cholangiopancreatography (ERCP) is restricted to cases with obstruction of the bile duct because is associated with significant adverse effects and has low profitability of achieving a histological diagnosis (20%) [7]. Endoscopic ultrasound (EUS) is an important complementary examination in the diagnosis and staging of PDAC as it permits examining the primary tumors, relationship with neighboring structures as well as obtaining tissue for pathological diagnoses [19]. The sensitivity and specificity of EUS-guided tumor fine needle aspiration (FNA) are 90 and 98%, respectively [20]. It should be noted, however, that patients with clinical and radiological suspected PDAC with resectable do not need a pathological diagnoses before surgical resection (IIIB). Pathological diagnoses are needed in patients with atypical presentation and in those with locally advanced disease that are managed with chemotherapy and/or radiation therapy (IIIB).

## Pathological diagnoses and classification

In the presence of suspicious lesions that are resectable, a tumor biopsy prior surgery is not required. Likewise, because of the complexity to obtain it as well as the limitations on its interpretation an intraoperative biopsy is not required [7, 21] (IIIB). A pathological diagnosis is always required in patients with unresectable or borderline lesions to be managed with chemotherapy and/or radiation therapy as well as in patients with amenable to treatment with metastatic disease [21] (IIIB).

The technique of choice to obtain tissue for pathological diagnoses depends on the location of the lesion and the stage of the disease. For primary tumors, a EUS-FNA is the safest procedure with highest sensitivity and specificity that can also provide additional staging information [22]. In patients with metastatic liver disease, a percutaneous US- or CT-guided biopsy is the procedure of choice [7, 21] (IIIB). If a biopsy does not confirm malignancy, it should be repeated atleast once. If during surgery the tumor is unresectable, histological diagnosis should be made (IIIB).

Table 1 summarizes the histopathologic classification of PDAC tumors. The most common type (95%) is ductal adenocarcinoma of the pancreas, referred to as pancreatic cancer. These tumors originate in ductal epithelium, have glandular differentiation, may produce mucin, and are associated with a significant desmoplastic reaction. There are no specific diagnostic markers to differentiate PDAC from other adenocarcinomas though reactive glands, a CK7+/CK20− profile, while not specific, supports a pancreatic origin.

**Table 1:** Pathological diagnosis

Classification of pancreatic tumors (WHO 2010 classification)
*Benign*
Acinar cell cystadenoma
Serous cystadenoma
*Premalignant lesions*
Pancreatic intraepithelial neoplasia type 3 (PanIN-3)
Intraductal papillary mucinous neoplasm with low or intermediate grade dysplasia
Intraductal papillary mucinous neoplasms
High-grade dysplasia
Tubulo-papillary intraductal neoplasia
Mucinous cystic dysplasia neoplasia with low or intermediate grade
Mucinous cystic neoplasm with high-grade dysplasia
*Malignant*
Ductal adenocarcinoma
Adenosquamous carcinoma
Colloid carcinoma (non-cystic mucinous carcinoma)
Hepatoid carcinoma
Medullary carcinoma
Cell carcinoma signet ring
Undifferentiated carcinoma
Undifferentiated carcinoma with osteoclast-like giant cells
Acinar cell carcinoma
Cystoadenocarcinoma acinar cells
Intraductal papillary mucinous neoplasm or associated with invasive carcinoma
Mixed acinar-ductal carcinoma
Mixed acinar-neuroendocrine carcinoma
Mixed-neuroendocrine carcinoma acinar-ductal
Mixed ductal-neuroendocrine carcinoma
Mucinous cystic neoplasm associated with invasive carcinoma
Pancreatoblastoma
Serous cystoadenocarcinoma
Pseudopapillary or solid neoplasia
Neuroendocrine neoplasms
Pancreatic neuroendocrine microadenoma
Neuroendocrine tumor (NET)
Pancreatic, not functioning G1, G2 NET
G1 NET
G2 NET
Neuroendocrine carcinoma (NEC)
Large cell NEC
Small cell NEC
NET serotonin producer (carcinoid)
Gastrinoma
Glucagonoma
Insulinoma
Somatostatinoma
VIPoma
Mature teratoma
Mesenchymal tumors
Lymphomas
Metastasis

## Biomarkers

Although many biomarkers have been studied for PDAC, only carbohydrate antigen 19.9 (CA 19.9) has proven useful and therefore, it is the only biomarker routinely used [23] (IA). For diagnoses, it has a sensitivity ranging between 70 and 92% and a specificity of 68–92% depending on tumor size. False positive results are associated with benign diseases such as pancreatitis, cirrhosis, acute cholangitis, and other diseases causing of cholestasis. Determining CA 19.9 is a complementary test in the diagnoses and management of PDAC [24]. In patients with resectable disease, plasma levels >100 U/mL values predict the presence of occult metastatic disease. In advanced disease, elevated CA 19.9 is considered as unfavorable prognostic factor [25, 26]. However, given its low positive predictive value, particularly in asymptomatic individuals, is not recommended as a screening marker [27].

## Timeline for diagnosis and management

Because PDAC is very aggressive, it is recommended to reduce the diagnostic time as much as possible to rapidly initiate treatment [28]. In a study in patients with advanced PDAC it was estimated that the average doubling time of pancreatic tumor was 40–60 days [29]. A month from the onset of the first symptoms or suspicious signs is a reasonable goal (IIIB).

The National Cancer Strategy Health System (2010) states that the time from therapeutic decision until the actual start of treatment should be less than 2 weeks for surgical treatment, one week for chemotherapy and 4 weeks (including treatment planning) for radiation therapy [30] (IIIB). Strategies to minimize time to diagnoses include raising awareness in the general population and among health professionals, rapid diagnostic protocols for patients with suspected lesion in primary care settings as well as preferential referral pathways to specialize care and treatment centers. PDAC, like any other complex tumor, should be managed in high referral centers by multidisciplinary teams: Medical oncologists, pathologists, radiotherapist, GI specialist, surgeons and radiologists [30] (IIIB).

## Staging and disease classification

Pancreatic cancer can be staged, based on imaging and pathological studies, in stages as per the TNM classification [31, 32]. However, from a management perspective, patients are better classified based on the extension of disease in resectable, borderline resectable, locally advanced unresectable and metastatic (IIIA).

In addition, from a treatment perspective, patients with locally advanced/metastatic disease are further classified as: (a) candidate to chemotherapy treatment without limitations; (b) candidate for chemotherapy with limitations and (c) not candidate for treatment with chemotherapy (Table 2) [6, 33–37] (IIIB).

**Table 2:** Patients’ classification, according to treatment perspective (IIIB)

Patients’ classification	Factors
Patient suitable for chemotherapy treatment without limitations	The presence of ALL the following factors ECOG 0–1 Age ≤75 years Bilirubin ≤1.5 ULN Good nutritional status (serum albumin >2.5 mg/dl, weight lost <10% over the last 3–6 months and BMI >20 kg/m^2^) Lack of co-morbidities
Patient suitable for chemotherapy with limitations	The presence of AT LEAST ONE of the following factors ECOG 2 (which can lead to KPS 70%) Age >75 years Mild to moderate neurological or endocrine-metabolic organ dysfunction; in case of liver dysfunction, hyperbilirubinemia >1.5 × ULN (once optimized if obstructive causes are present, for example with biliary stent) marks the degree of dysfunction. It is considered appropriate to adjust the dose, for example, using GEM at 600-800 mg/m^2^ and nab paclitaxel 75–100 mg/m^2^) [37] Cardiac dysfunction, especially a recent ischemic event; acute, symptomatic, severe TED such as PE with hemodynamic instability or DVT with risk and limb amputation [38] BMI <20 kg/m^2^ or >10% weight loss in 3–6 months
Patient not suitable for chemotherapy treatment	The presence of AT LEAST ONE of the following factors ECOG 3-4 (which may result in KPS ≤ 60%). Active treatment will be initiated in patients with ECOG 3 secondary to the disease (not to their previous comorbidities) without any severe organ dysfunction, thus moving this subgroup of patients to the “candidate for chemotherapy treatment with limitations” group Severe organ dysfunction: neurological (e.g., severe cognitive impairment, Alzheimer’s type); endocrine-metabolic, infectious (uncontrolled HIV), renal, hepatic dysfunctions, etc

*ECOG* Eastern Cooperative Oncology Group, *ULN* upper normal limit, *BMI* body mass index, *GEM* gemcitabine, *KPS* Karnofsky performance status, *TED* thromboembolic disease, *PE* pulmonary embolism, *DVT* deep venous thrombosis

## Practical considerations in treatment decision process

The treatment plans for patients with PDAC patients should be made individually. A complete staging process is essential to determine the extent of the tumor that drives treatment plan and prognosis. In parallel, patient´s status, which is linked to its ability to tolerate an aggressive treatment, should be defined. This includes the functional status as determine by the Karnofsky Performance Scale (KPS) and/or the Eastern Cooperative Oncology Group (ECOG). Patients with KPS of less than 60–70% or ECOG less than 0–1 are limited to receive aggressive chemotherapy. For elderly patients, it is also advisable to use geriatric scales such as the Barthel scale that assesses the degree of autonomy in basic activities of daily living [37]. The assessment of nutritional status as measured by physical exam (weight, body mass index, presence of edema), recent weight lost (>10% over 6 months); plasma protein levels (albumin, prealbumin, transferrin) is crucial [38]. Validated nutritional scales such as Mini Nutritional Assessment are useful in this regard. In addition, a life expectancy of >3 months is usually needed to administer cancer treatment. Mechanical problems caused by tumor masses such as bile duct and bowel obstruction need to be assessed and corrected prior to treatment commencement. Finally, patient priorities and preferences need to be considered (IIIA).

## Treatment approaches

### Resectable disease/borderline resectable disease

#### Neoadjuvant treatment

Neoadjuvant treatment, which is the treatment with chemotherapy and/or radiotherapy administered before surgical resections, aims to increase overall survival by increasing the rate of R0 resection and early treatment of micrometastatic disease. In addition, preoperative treatment may lead to avoiding unnecessary surgical resection in patients with aggressive tumors that develop early progression.

It should be noted, however, that there are no randomized phase 3 studies to support any of these assumptions. Prior studies suggest an increment in the rate of R0 resections [39–41]. Most studies reported thus far were conducted with old, less effective chemotherapy regiments and the data available with modern regimens [gemcitabine (GEM)/nab paclitaxel, FOLFIRINOX], came from single-center trials [35, 42–47].

Here, we discuss preoperative management of patients with resectable or borderline resectable disease. Prior to treatment initiation, it is important to have pathological diagnosis as well as normalized bile duct drainage. Endoscopically placement of a metal stent is the procedure of choice to palliate obstructive jaundice (IIIB).

For patients with resectable disease neoadjuvant treatment cannot be recommended outside a clinical trial. However, preoperative treatment is one of the available approaches in patients with borderline resectable disease (IIB). The chemotherapy treatments used should be those associated with higher response rate in patients with metastatic disease (GEM/nab paclitaxel, FOLFIRINOX) [35, 46] (IIB). Currently there is no evidence to recommend one versus the other and the decision should be based on patients´ characteristics and center experience. In general, treatment should be administered for 3–4 months with a reassessment and multidisciplinary discussion afterwards (IIB). Patients with responding tumors by either radiological criteria or CA 19.9 could proceed to surgical resection [48, 49] (IIB). Radiotherapy the alone is not recommended and should be combined with either fluoropyrimidines or GEM (IIB). IMRT is associated with reduced toxicity and should be used when available. Patients who receive chemo-radiation should wait four to eight weeks before attempting surgical resection (IIB).

Radiological evaluation must be conducted after neoadjuvant treatment. Lack of objective radiological response should not be a criterion to rule out surgical resection [52] (IIB). Those patients with suspected disease progression by either elevated CA 19.9 without radiological evidence of disease progression should be carefully evaluated and PET scan and laparoscopy should be considered (IIB). Patients with documented metastatic progression are not candidates for surgery and should be managed as such (IIB).

#### Surgical treatment

An R0 surgical resection is the only curative treatment for patients with pancreas cancer and should always be attempted. Prior to considering surgery, patients need to be assessed by a multidisciplinary team and classified as resectable, borderline resectable or unresectable locally advanced being the multidetector CT scan the radiological procedure of choice for this matter [18, 50] (IIA). Based on the extent of the tumor, involvement of blood vessels [portal vein, superior mesenteric vein (SMV); superior mesenteric artery (SMA); celiac trunk and hepatic artery] patients are classified in one of the above-mentioned group [31, 51–55]. Table 3 provides the specific criteria [57]. More recent classifications also include changes induced by preoperative treatments. It should be noted that extension to adjacent organs, if resectable, is not a contraindication for surgery.

**Table 3:** Resectability criteria [57]

Category	Arterial	Venous
Resectable	Absence of tumoral contact with CT, MSA or CHA	Absence of tumoral contact with SMV or PV or contact ≤180° without irregularities in the venous contour
Borderline resectable	Head of the pancreas and uncinate process Solid tumoral contact with CHA, without extension to CT or HA bifurcation, that allows resection and complete and safe vascular reconstruction Solid tumoral contact with SMA ≤180° The presence of arterial anatomic variants should be evaluated (i.e., right accessory HA, replacement of right HA, replacement of CHA as well as source of replaced or accessory artery) and the presence and degree of tumoral contact due to their influence when planning the surgical procedure Body and tail of the pancreas Solid tumoral contact with CT ≤ 180° Solid tumoral contact with CT > 180° without aorta involvement and with intact GDA (there is no consensus on this criteria and can be included in the non-resectable category)	Solid tumoral contact with SMV or PV >180°, contact ≤180° with irregularities in the venous contour or venous thrombosis but with adequate proximal and distal ends that allow safe vascular resection and replacement Solid tumor contact with IVC
Non-resectable	Distant metastasis (including metastasis in non-regional lymph nodes) Head of the pancreas and uncinate process Solid tumoral contact with SMA or CT >180° Solid tumoral contact with the first jejunal branch of SMA Body and tail of the pancreas Solid tumoral contact with SMA or CTC > 180° Solid tumoral contact with CT and aortic infiltration	Head of the pancreas and uncinate process Tumoral infiltration or thrombosis (thrombosis may not be tumoral) in SMV or PV, that does not allow reconstruction Contact with the most proximal jejunal vein that drains in SMV Body and tail of the pancreas Tumoral infiltration or thrombosis (the thrombosis may not be tumoral) in SMV or PV, that does not allow reconstruction

*CT* celiac trunk, *SMA* superior mesenteric artery, *CHA* common hepatic artery, *SMV* superior mesenteric vein, *PV* portal vein, *IVC* inferior vena cava inferior, *GDA* gastroduodenal artery

In addition to stage classifications, a complete assessment of operative risk should be performed. Considering the high morbimortality of pancreatic cancer resection, its assessment is of great importance. Classic surgical risk scales, such as Apache, ASA and POSSUM, do not predict accurately the morbidity after pancreatic surgery. Other more recent classification such as the one published by Braga, as well as the Preoperative Pancreatic Resection (PREPARE) and SOAR (Surgical Outcomes Analysis and Research) scores are based on the integration of multiple parameters and appear more accurate [56–59].

Prior to surgical resection, it is critical to gain an adequate nutritional status and either nutritional supplements or even parenteral nutrition should be considered for 1–2 weeks prior to surgery in malnourished patients. In patients with large tumors, particularly of the tail of the pancreas, borderline resection and high tumor marker, a diagnostic laparoscopy should be considered prior to laparotomy.

For patients with tumors in the head of the pancreas, the procedure of choice is the duodenal pancreatectomy (Whipple procedure), which includes en bloc resection of the head of the pancreas, duodenum, gall bladder and bile duct, together with regional lymphadenectomy [57] (IA). Pylorus preserving pancreatectomy is equivalent to classic Whipple with regards to morbimortality and outcome and the selection of surgery type depends on surgeon preference. Other procedures such as extended pancreatectomy, total pancreatectomy, and extended lymphadenectomy are reserved for selected cases [60, 61].

Patients with tumors of the body or tail of the pancreas are treated with distal pancreatectomy.

As mentioned above, laparoscopy can detect small peritoneal implants or liver metastasis not visible by CT scan and is often used in patients with high risk of metastatic disease [62] (IIB). In addition, for patients with tumors in the body and tail of the pan, laparoscopy resection with or without robot assistance, is gaining acceptance [63]. Finally, vascular involvement has been traditionally considered a formal contraindication for resection [64, 65]. More recently, however, venous resection and reconstruction is accepted as an optimal surgical procedure and is not associated with worse prognosis. However, arterial resection and reconstruction is still considered an investigational approach [51, 57, 65].

#### Adjuvant treatment

Adjuvant treatment is recommended in patients who undergo an R0/R1 resection with a PT1-4/N0-1M0, with an ECOG PS 0–1 and proper nutritional status [66, 67]. Treatment of patients with ECOG 2 needs to be individualized [66, 67].

It is recommended that adjuvant treatment is initiated within the next 12 weeks after surgery in patients who do not have any active infection, any serious postsurgical complication or presents with signs or symptoms of recurrent disease. There is no consensus on the adjuvant treatment in patients who have received neoadjuvant treatment [7, 68–70]. Those patients need to be evaluated in a multidisciplinary board. In general, adjuvant treatment in this population is still considered investigational. As a general rule, patients who have received neoadjuvant treatment should receive adjuvant treatment to complete a total of six months of treatment (IIIB).

With regards to the role of radiation therapy, there is even less information and could be considered in those patients with positive margin providing was not administered in the preoperative period (IIC). Prior to adjuvant chemotherapy commencement patient needs to be evaluated with a CBC, chemistry, renal function test, albumin, LDH and CA 19.9 levels [7, 71–73]. A CT of the chest, abdomen and pelvis is required to document lack of disease progression [18, 71, 73]. Currently, and until the results of ongoing studies are available, the recommended treatment is single agent GEM, or 5-FU and leucovorin (LV), for a total of six months [66, 67, 74–76] (IA). The results of the recent clinical trial ESPAC-4 support the use of GEM + capecitabine in this setting [76] (IA). The role of radiation therapy is less defined and should be considered for patients with positive margins and in selected cases with lymph node positive disease [75–82] (IC).

Those patients with positive margins and neoadjuvant treatment that did not include radiation therapy are a particularly suitable group for postoperative radiotherapy. Currently, there is no biomarker predictive of outcome in this patient population and CA 19.9 is the only prognostic indicator [83]. Once treatment is completed, patient should be followed every three months with measurement of CA 19.9 levels and a physical exam [7, 83–85] (IIIB). A CT scan should be performed every 6 months during the first 2–3 years after surgery and yearly thereafter [7, 83–85].

### Unresectable disease

#### Management of patients with locally advanced disease

The management of patients with locally advanced disease is one of the most controversial areas in the treatment of PDAC due to the paucity of well controlled, randomized clinical trials. The goal in treating these patients is to improve survival which is better achieved if a complete surgical resection is feasible. Patients with locally advanced disease need to be evaluated like any other patient with PDAC with special attention to nutritional status, ECOG performance status, symptoms related to tumor local growth (pain, bowel and/or bile duct obstruction). The presence of bile duct or bowel obstruction need to be corrected before treatment is initiated. For patients who are candidates of chemotherapy treatment without limitations, this is in general the recommended approach (Table 2). While there are no data with regards to the most efficient regimen in this particular setting, current trend is to use either GEM-nab paclitaxel or FOLFILRINOX based on the data available for patients with advanced disease [35, 46, 86, 87] (IIB). Chemotherapy is usually administered for 3–4 months followed by assessment of tumor response. In patients with partial response that allows surgical resection, it could be a treatment option. In the remaining patients with partial response and those with stable disease, chemotherapy treatment as well as consolidation with chemotherapy and radiotherapy are valid options.

The recent data from the LAP007 study (Phase III study that compared chemoradiotherapy and chemotherapy in patients with a locally advanced pancreatic cancer controlled after four months of GEM with or without erlotinib) indicates that there are no survival benefits for chemo-radiation as compared to continuing chemotherapy alone, although it decreased the risk of local progression and improved PFS [88] (IA).

It should be noted, however, that this study used conventional external beam radiation and gemcitabine-based chemotherapy and the results cannot be extrapolated to those obtained with new chemotherapy regimens and more modern radiation techniques (IMRT). Data from the SCALOP trial (Phase II study of induction chemotherapy followed by GEM or capecitabine-based chemoradiation in locally advanced pancreatic cancer) suggest that the radiotherapy combined with fluoropyrimidines achieves better results than when combined with GEM [89] (IA). Patients who are candidates for chemotherapy with limitations have a very poor prognosis and should be managed with either single agent chemotherapy (GEM alone), combination therapy (GEM + nab paclitaxel) or radiation therapy alone (Table 2).

#### Management of patients with metastatic disease

*First line treatment* The management of patients with advanced PDAC is based on systemic chemotherapy. In those patients who have received prior adjuvant or neadjuvant treatment, rechallenge should be considered when the disease-free interval is ≥6 months. Prior to treatment initiation, patients should be classified based on performance status, nutritional status, age and comorbidities according to Table 2. For patients able to receive chemotherapy without limitations, the current standard of care is either GEM-nab paclitaxel or FOLFIRINOX [35, 46] (IA). In the lack of randomized studies comparing these two regiments no one can be recommended [90, 91]. FOLFIRINOX should not be administered to patients >75 years old. In addition, this regimen is associated with a higher incidence of toxicity and thromboembolic complications and often requires growth factor support. FOLFIRINOX should be used with caution in patients with biliary stents who have increased risk of biliary tract infections and sepsis. In addition, it is recommended to administer antiemetic prophylaxis at least for moderately emetogenic chemotherapy. Patients who are candidates to chemotherapy with limitations are best managed with GEM-nab paclitaxel, and should be administered until progression or unacceptable toxicity; this is particularly the case for patients with ECOG 2 secondary to high tumor volume in whom tumor reduction may result in symptomatic improvement (IIB). Patients with ECOG 2 secondary to comorbidities or those with severe peripheral neuropathy can be treated with GEM alone (IIIB). Patients who are not candidates for chemotherapy should receive palliative treatment. The optimal management should always be reassessed and modified if the condition of the patient changes. Thus, subjects whose condition improves should be considered for more aggressive approaches. In any group, enrollment in a clinical trial should always be the preferred option (Algorithm 2).

*Second line treatment* Second line treatment is in general recommended after progression to first line treatment [92] (IA). Treatment decision should be based on the general status of the patient as well as the first line treatment. For patients who have been treated with GEM based regimen FOLFOX chemotherapy has demonstrated improvement in survival as compared to 5-FU in the CONKO-003 study [93]. These results, however, have not been confirmed in the PANCREOX trial [94]. More recently, the NAPOLI-1 showed that MM-398 (liposomal formulation of irinotecan) in combination with 5-FU/LV is better than 5-FU/LV alone [95]. For patients who have received 5-FU/LV based chemotherapy on the first lie sett, there is very little data to base second line choices. In general, either GEM alone or GEM combination is recommended [96].

*Treatment monitoring* The response to treatment should be monitored every 8–12 weeks by a CT scan of the chest, abdomen and pelvis (IIIB). Other imaging modalities such as MRI and/or PET are not routinely recommended to be used in a serial basis (IIIB). The tumor marker CA 19.9 should be measured before treatment and every 4–8 weeks thereafter (IIIB). Tumor progression in patients with rising CA 19.9 should be confirmed radiologically [7, 35, 46, 97] (IIIB).

## Supportive care

Supportive care aims to improve symptoms, reduce hospital admission and preserve quality of life. Proper symptomatic management is critical to allow administration of chemotherapy and radiotherapy. Symptomatic management should be accomplished in a multidisciplinary fashion. In this section, we will describe the most common approach to diagnosis and management of the most common symptoms of PADC.

1. Bile duct obstruction.

Up to 75% of patients with tumors in the head of the pancreas develop bile duct obstruction which results in jaundice, itching cholangitis and hepatic dysfunction. If untreated, hepatic failure may ensure [98]. While surgical management had been the preferred approach in the past, particularly coledocoenterostomy, the high mortality of these techniques together with the excellent results obtained with endoscopic approaches has resulted in the preference of endoscopic management [99]. The preferred approach is endoscopic stent (via ERCP) [100]. Percutaneous transhepatic colangiopancreatography with stent placement is associated with higher risk of infectious complications and bleeding, being only recommended for patients in whom the endoscopic approach is not feasible [101]. Plastic stents have a life span of about four months and are only recommended for patients with expected short survival. Patients with longer expected survival should be treated with metal stents that have a longer functionality [102]. Surgical management is only recommended for patients who undergo a laparotomy for other reasons. Bile duct drainage is clearly recommended in patients who are scheduled for preoperative chemotherapy, those with cholangitis, or those in whom surgical resection is expected to be delayed. However, patients with moderate bilirubin elevation scheduled to undergo surgery can be safely operated without drainage [103–105].

2. Duodenal obstruction.

Ten to twenty-five per cent of patients develop duodenal obstruction which is associated with severe symptoms and deterioration of quality of life. The most common approach nowadays is the endoscopic placement of an expansible metallic stent. This approach results in over 90% success with very few complications. The preferred surgical treatment is a gastric jejunonostomy which is only recommended in very selected patients because of high morbidity and mortality when performed as a treatment modality [106]. Prophylactic gastrojejunostomy should be considered in patients with non-resectable tumors who undergo an exploratory laparotomy. Endoscopic stents are associated with rapid recovery of oral intake, less morbimortality and a shorter hospital stay [107, 108]. In contrast, surgical treatment is associated with better long term outcome. For this reason surgery is only considered for patients with expected long survival. All these recommendations achieve a level of evidence IIB.

3. Pain.

Fifty to sixty per cent of patients with PADC develop some short of pain. These patients need intensive treatment, with both pharmacological and non-pharmacological approaches [109]. It is important to consider the precise cause of the pain, such as for example bowel obstruction, liver or bone metastasis, or secondary to chemotherapy (neuropathy, mucositis, enteritis) [110–113]. Table 4 summarizes the most important approaches for pain management (IIA).

**Table 4:** Pain management strategies (IIA)

Mild pain	NSAIDs: Taking into account maximum doses and side effects (gastrointestinal bleeding, nephrotoxicity [110] acetaminophen
Moderate/severe pain	Opioids: any opioid as first choice, except for methadone (secondary choice). Methadone has great benefit in neuropathic pain due to its anti NMDA effect. Should be administered by trained personnel
Other treatments	
Adjuvant treatment/co-analgesics	Corticosteroids Gabapentine (if neuropathic pain)
Intrathecal catheters	To manage moderate to severe pain Lower frequency of secondary adverse events, with better pain control [111] First choice: hydromorphone, ziconotide, local anesthetic Severe neuropathic pain: baclofen, clonidine
Miscellaneous (little evidence)	Other therapies: phentolamine, capsaicin, cryoablation, acupuncture
Radiotherapy	Indicated for management of refractory pain, especially in patients with good performance status and localized pain caused by isolated metastases or pancreas and adjacent structures involvement
Celiac plexus block [112]	Provides better analgesic control (benefit in >80% of the patients) and/or decreases the opioid dose when compared to standard analgesic treatment Cause a disruption in the pain signaling by an average of 3 months It can be performed via percutaneous under ultrasound control, surgical or endoscopic by ultrasound. In terms of technique, there isn’t enough evidence to make any recommendations Side effects are rare (transient hypotension, constipation or diarrhea) There is no evidence to recommend the timing for the blockage (early, at diagnosis, or late when there is poor pain control) There is no evidence that increases survival In the clinical practice celiac plexus block is reserved for patients with poor pain control despite escalation with opioids or for those with opioid related secondary adverse events [113] There are limited data regarding the repeated use of celiac plexus block (pain relief is achieved in 29% of the patients)

4. Nutritional support.

Malnourishment is very common in patients with PADC secondary to problems with intake as well as cancer-associated cachexia. Frequent assessment of nutritional status is recommended, being Patient-Generated Subjective Global Assessment (PG-SGA) the most common scale used in oncology [114]. Intervention ranges from dietary counseling, dietary supplements and enteral feeding. Parenteral nutrition is only recommended as a temporary approach in patients with transient inability for enteral feeding with good general status. For patients scheduled to undergo surgery who present with severe malnutrition (weight loss >10% in 6 months, BMI <18.5 kg/m^2^ and serum albumin <3 g/dl) it is recommended to provide nutritional support by dietary supplements, enteral feeding or parenteral feeding for 7–14 days. More than 50% with PADC have pancreatic exocrine insufficiency. This problem usually presents as esteatorrhea. Optimal substitutive treatment with substitute pancreatic enzymes is recommended (25,000–150,000 units per meal intake) [115].

Cachexia is a multifactorial syndrome characterized by permanent loss of lean body mass. It does not respond to conventional nutritional support and leads to progressive functional deterioration. 20 to 80% of patients with PADC have cachexia, being more common in advance disease [116]. It is a poor prognosis factor and unfortunately, there is no effective treatment once established. Identification of patients in a pre-cachexia stage were multimodality approach may reverse the symptoms is critical [117]. Megestrol acetate and high dose steroids are approved for this condition. However, in case of megestrol acetate, side effects such as thrombotic episodes limit its universal recommendation [118]. All these nutritional support recommendations achieve a level of evidence of IIB.

5. Thromboembolic disease.

Thromboembolic disease (TED) is one of the most common complications, with an incidence of 20–35% [119]. Its etiology is multifactorial, being associated with poor prognosis, particularly in patients with early thrombosis. The risk of TED increase in the perioperative period in patients with advanced disease and in those treated with chemotherapy. An elevated D-dymer, poor performance status, central catheter and absence of prophylaxis increases the risk. The Khorana index is useful to identify high risk patients [120]. In randomized clinical trials, prophylaxis of deep venous thrombosis (DVT) in patients with PDAC resulted in significant decrease in the rate of thrombosis, with no impact on survival [121, 122]. Routine prophylaxis of thrombosis is not recommended in the ambulatory setting [123] (IA). In patients with a Khorana index ≥3 and no risk of bleeding, prophylaxis with low molecular weight heparin could be considered (IIB). Hospitalized patients and those who underwent surgery treatment with low molecular weight heparin are recommended for at least 6 months (IIA).
